# Malaria in Angola: recent progress, challenges and future opportunities using parasite demography studies

**DOI:** 10.1186/s12936-022-04424-y

**Published:** 2022-12-28

**Authors:** Wilson Tavares, Joana Morais, José F. Martins, Ryan J. Scalsky, Thomas C. Stabler, Márcia M. Medeiros, Filomeno J. Fortes, Ana Paula Arez, Joana C. Silva

**Affiliations:** 1 Global Health and Tropical Medicine, GHTM, Instituto de Higiene E Medicina Tropical, IHMT, Universidade NOVA de Lisboa, UNL, Lisbon, Portugal; 2 Instituto Nacional de Investigação Em Saúde, INIS, Luanda, Angola; 3 Programa Nacional de Controlo da Malária, PNCM, Luanda, Angola; 4 Institute for Genome Sciences, University of Maryland School of Medicine, Baltimore, USA; 5 Department of Medical Parasitology and Infection Biology, Swiss Tropical and Public Health Institute, Basel, Switzerland; 6 University of Basel, Basel, Switzerland; 7 Department of Microbiology and Immunology, University of Maryland School of Medicine, Baltimore, USA

**Keywords:** Malaria, Angola, *Plasmodium falciparum*, Elimination eight regional initiative

## Abstract

Over the past two decades, a considerable expansion of malaria interventions has occurred at the national level in Angola, together with cross-border initiatives and regional efforts in southern Africa. Currently, Angola aims to consolidate malaria control and to accelerate the transition from control to pre-elimination, along with other country members of the Elimination 8 initiative. However, the tremendous heterogeneity in malaria prevalence among Angolan provinces, as well as internal population movements and migration across borders, represent major challenges for the Angolan National Malaria Control Programme. This review aims to contribute to the understanding of factors underlying the complex malaria situation in Angola and to encourage future research studies on transmission dynamics and population structure of *Plasmodium falciparum*, important areas to complement host epidemiological information and to help reenergize the goal of malaria elimination in the country.

## Background

Malaria is a vector-borne disease caused by *Plasmodium* parasites and transmitted through the bite of infected female mosquitoes from the genus *Anopheles*. *Plasmodium falciparum* is the deadliest human malaria parasite and the most prevalent species in sub-Saharan Africa [1]. According to the World Health Organization (WHO), the estimated number of global malaria deaths increased from 558,000 in 2019 to 627,000 in 2020, due in part to moderate disruptions in the delivery of malaria services during the COVID-19 pandemic [1–5].

Understanding the challenges to malaria control and elimination in countries where its prevalence is highest is critical to achieve the eventual eradication of malaria, as is the continued implementation of effective strategies of parasite monitoring and control that have fueled recent progress. The aims of the present review are (*i*) to contribute to the understanding of the factors associated with the complexity of malaria in Angola, which ranked fifth and nineth among African countries in number of malaria cases and deaths, respectively, in 2020 [1], and (*ii*) to summarize some key topics where research on genomic epidemiology of *P. falciparum* can contribute to sustain the progress in malaria control and eventual elimination in the country [6–9].

Malaria is a major public health concern and poses the biggest health threat to pregnant women and children under five in malaria-endemic countries such as Angola [10]. As of 2018, malaria, primarily caused by *P. falciparum*, remained among the top causes of mortality from infectious diseases in Angola, together with HIV/AIDS and tuberculosis [11, 12]. Malaria prevalence among children under 5 years of age decreased by 38% between 2006 and 2011 [13], although in 2011 malaria was still responsible for 35% of all treatment demand, 20% of all hospitalizations, 40% of prenatal mortality, 25% of maternal mortality, 60% of hospitalizations in children under 5 and 35% of infant mortality [13]. However, the country has achieved tremendous progress in the past two decades in ameliorating the impacts of malaria. The mortality rate from malaria has dropped by an estimated 36% since 2000 [10], and population-wide morbidity rates due to the disease decreased from 26.6% in 2000 to 15% for 2018 [11, 13].

Angola has a highly diverse climate and varied ecosystems and, similarly, malaria prevalence and mosquito vectors are distributed heterogeneously across the country. Provinces of hyperendemicity (malaria incidence ≥ 30%) are located in the rainier, warmer north and northeast provinces, while the temperate central and coastal provinces have low to mesoendemic (10%  < malaria incidence < 30%), stable transmission; finally, the southern, arid regions closest to Namibia have highly seasonal (mesoendemic, unstable) malaria transmission and are prone to epidemics (malaria incidence ≤ 10%) [14–16]. Based on available annual malaria surveillance reports, malaria outbreaks have been occurring in several central-coastal provinces since 2015, with the highest numbers reported in 2016 and 2017 [17]. This wide range in malaria transmission intensity necessitates different approaches to, and priorities in, malaria control depending on the region [18]. Despite the broad range in transmission intensity across the country, the entire Angolan population remains at risk for malaria infection [10].

The control of malaria in Angola is not only critical for the country itself, but for its neighbours as well. Angola’s geographical location encompasses the southwestern-most edge of the African continent’s region of malaria endemicity. Malaria is endemic and highly prevalent among Angola’s neighbours to the north and east, but to the south, in northern Namibia, it is rarer and occurs mostly as periodic epidemics. Angola belongs to the group of eight nations in southern Africa aiming to eliminate malaria by 2030, as part of the Malaria Elimination Eight Regional Initiative, E8 [19]. In addition to the Government of Angola, the study and control of malaria in the country is supported principally by the United States Agency for International Development (through its U.S. President’s Malaria Initiative, USAID/PMI), among other international partners, with the goal of strengthening the country’s strategic cooperation and partnership with E8, to consolidate the continent’s elimination goal in the West-Southern African countries [16, 20, 21]. Recent efforts have focused on the characterization of cross-border malaria transmission between Angola and Namibia [22–24]. However, there are currently no studies that place *Plasmodium* populations from Angola in the broader context of malaria in central and southern Africa, to inform the intensity and directionality of parasite migration between countries.

This review provides greater detail regarding some of Angola’s challenges to control malaria, including joint efforts with neighbouring countries, and offers a perspective of incorporating genomic studies to better understand these complex challenges to further progress towards Angola’s goal of malaria elimination.

## Angola’s variable malaria incidence rates and factors that contribute to stratification

### Epidemiological stratification and incidence rates of malaria in Angola

**Fig. 1:**
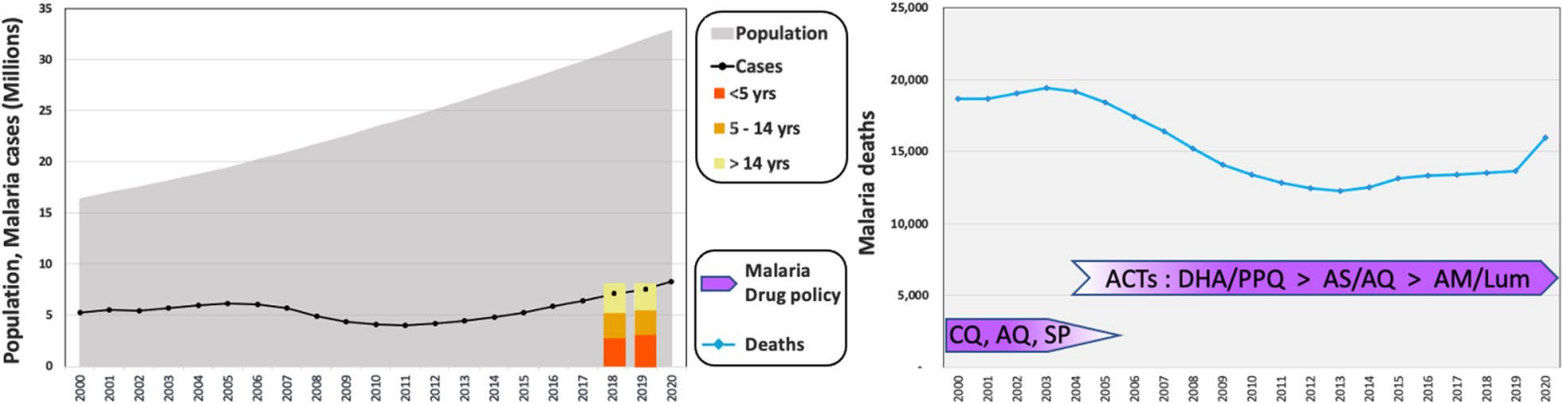
Angola’s malaria statistics. Angola has seen a doubling in population since the turn of the century (grey, left axis), while the total number of cases (black line; left axis) has remained relatively constant in the same period and the number of deaths decreased by close to a third (blue line; right axis). Recent statistics from Angola’s NMCP show malaria cases roughly equally distributed among age classes (yellow-orange). The years of 2004–2008 marked a transition of drug policy from non-ACTs to artemisinin-based combination therapy (ACT) (purple), concomitant with a decrease in total malaria deaths and a slight decrease in total cases, despite rapid population growth

Angola is the 12th most populous African country, with close to 36 million people [25]. The country has a rapidly expanding population, which has doubled since 2000; the median age is 16.7 years, and close to a third (32.6%) of the population is under the age of 10 years [26]. Its world rank by population size is expected to change from 44th currently to 24th by 2050 [25]. The entire Angolan population is at risk for malaria, but overall incidence has gradually decreased. In 2020, Angola saw an estimated 7.5 million malaria cases and 13,600 deaths, compared to 5.3 million cases and 19,000 deaths in 2000, in a population that was half its current size [10] (Fig. 1).

In Angola, children under 5 years of age and pregnant women are the most vulnerable populations to malaria [27]. According to Angola’s National Malaria Control Programme (NMCP), in recent years, malaria prevalence was nearly equally distributed among three age groups: infants and children under the age of 5 years (35–39%), children 5 to 14 years old (31%–29%) and individuals older than 15 (32%–37%) (Fig. 1).

**Fig. 2:**
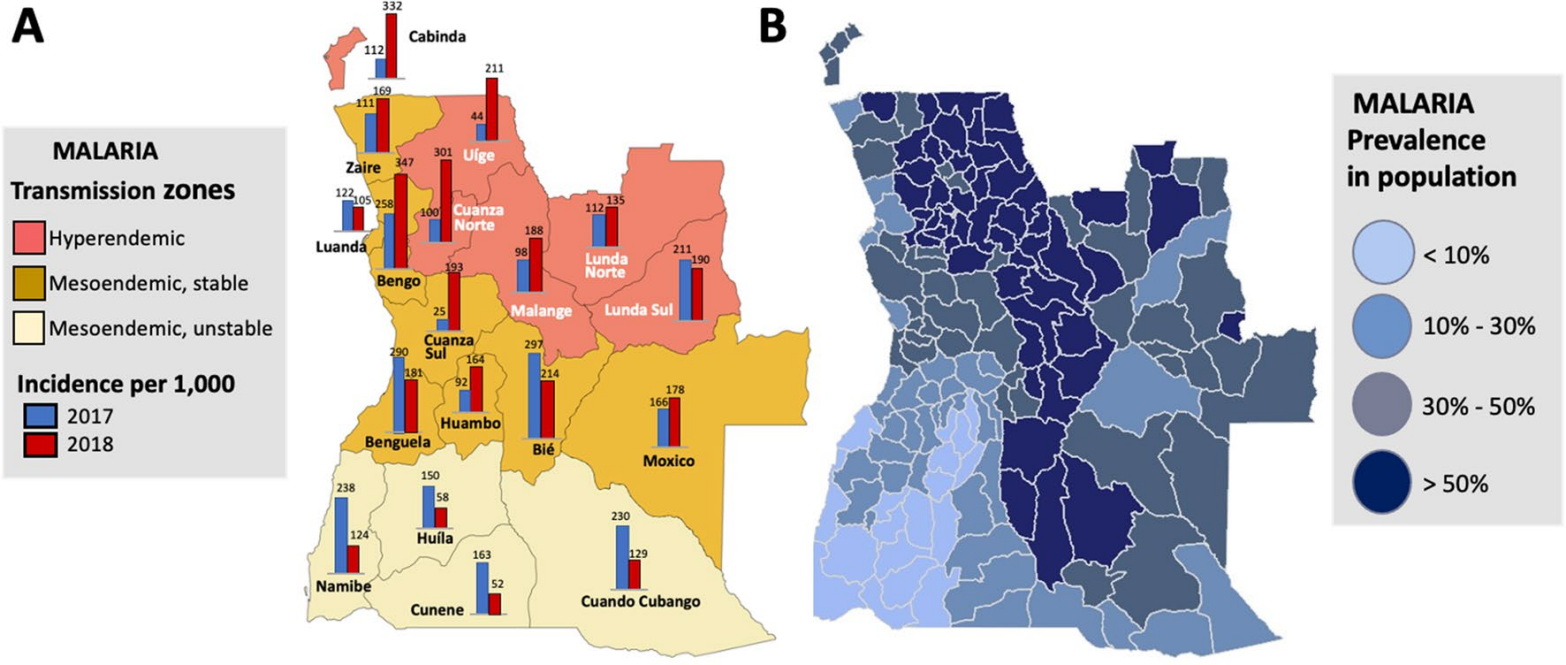
Malaria transmission in Angola. Angola’s provinces are categorized into three transmission zones, which differ by intensity and seasonal stability of transmission. **A** Malaria incidence rates (per 1000 individuals) in 2017 (blue) and 2018 (red), as reported by the Angolan NMCP are shown for each province [14]. **B** Malaria prevalence in the general population is shown at the municipal level, estimated from data collected between 2015 and 2018 (from [21])

The stratification of malaria burden in mainland Angola occurs in three distinct zones, closely associated with climate: (a) Equatorial Zone, reaching high temperatures and rainfall (October to May), and encompassing malaria-hyperendemic provinces with high transmission year round; (b) Tropical Zone, extending along the coast from the Congo basin to the province of Benguela and along the middle of country in a E-W axis, which can include malaria hyper- to mesoendemic regions; and (c) Sub-tropical Zone, corresponding to the southern region of Angola, influenced by considerably high thermal amplitudes near the Namib Desert, where regions of low and seasonal malaria are found [16, 28] (Fig. 2A).

According to the results obtained by the Angolan NMCP in 2018, the provinces of Cuanza Norte, Cabinda and Bengo, located in hyper- or meso-endemic regions, had the highest incidence rates, which increased significantly between 2017 and 2018 (Fig. 2A). In contrast, Cunene and Huíla, provinces with moderate unstable transmission, recorded the lowest incidence rates in 2018 (Fig. 2A). While incidence rates between 2017 and 2018 decreased in some central and coastal provinces, such as Bié and Benguela, the most dramatic reductions were seen in southern provinces bordering Namibia, from Cuando Cubango in the SE to Namibe in the SW [14, 15, 17]. The prevalence of malaria is roughly associated with transmission intensity zone; however, there can be dramatic variation in disease prevalence within each province (Fig. 2B) [21], which brings into question the robustness of the trends observed.

Despite decreases in new cases and incidence rate in several provinces, Angola continues to report outbreaks as a result of autochthonous spread and, in some areas, the burden of malaria has remained unchanged or increased. Luanda is one of the most vulnerable provinces to malaria epidemics, which are closely linked to high population density and inadequate sanitation [14, 15, 17]. Investments in housing programmes and implementing or strengthening a proactive surveillance system are some of the measures proposed to reduce the recurrence of malaria outbreaks [29]. However, it remains unclear what proportion of outbreaks are due to within-province transmission versus to cases imported from other provinces due to population migration.

The prevalence of malaria among children under 5 years of age differs among provinces, from ≤ 1% in the SW of the country, in the provinces of Namibe, Cunene, Huíla and Huambo, where transmission intensity is low, to 30–40% in the hyperendemic NW provinces of Uíge and Cuanza Norte, according to statistics from Angola’s 2016 Demographic and Health Survey reported by USAID/PMI [16]. Surprisingly, malaria prevalence among young children was also reported to be very high in the central and SE provinces of Cuando Cubango, Moxico and Bié, where average transmission intensity is thought to be relatively low and, conversely, the reported prevalence was relatively low in the province of Lunda Sul, where malaria is hyperendemic [16]. It remains unclear if these inconsistencies point to ongoing regional shifts in the patterns of malaria prevalence between 2016 and 2018, if they reflect epidemiological features of disease distribution in terms of incidence and prevalence across age groups, or are artifacts resulting from considerable within-province heterogeneity in malaria prevalence [16, 30]. However, the accuracy of malaria prevalence estimates in Angola has traditionally been somewhat compromised by incomplete reporting, inability to test due to stockouts of the necessary materials, and other logistic challenges, such as transportation of materials to remote areas and efficient distribution [15, 16, 31]. Additional challenges include mis- or over-diagnosis, due to factors such as the presence of diverse *Plasmodium* species, other fever-causing pathogens that can co-occur with malaria [31–33].

Future progress in malaria control and elimination in Angola depends on improvements in all those challenge areas. A panel of stakeholders, including representatives of the Angolan Ministry of Health, provincial governments, patient groups and United Nations agencies, was convened recently to summarize lessons learned after the first year of implementation of Global Fund-supported measures to control HIV, tuberculosis and malaria in two Angolan provinces [34]. The panel determined that recent progress was facilitated by the adoption of the health management information system DHIS2, but that key needs remain, including in logistics, such as improved communication and coordination, timely acquisition of supplies and implementation of reliable distribution chains, but, critically, in the recruitment and training of human resources [34]. This also extends to additional technical and clinical training needed for local health care workers, an issue that has plagued the Angolan heath system for years, but with marked improvements seen recently under a training programme launched by the PMI on malaria case management [31, 35, 36].

The dramatic differences in malaria incidence across provinces is due, in part, to variation in climate and topography in this vast country which, in turn, impact the distribution on the disease vector. These factors are addressed next.

### Angola’s diverse climate and topography

**Fig. 3:**
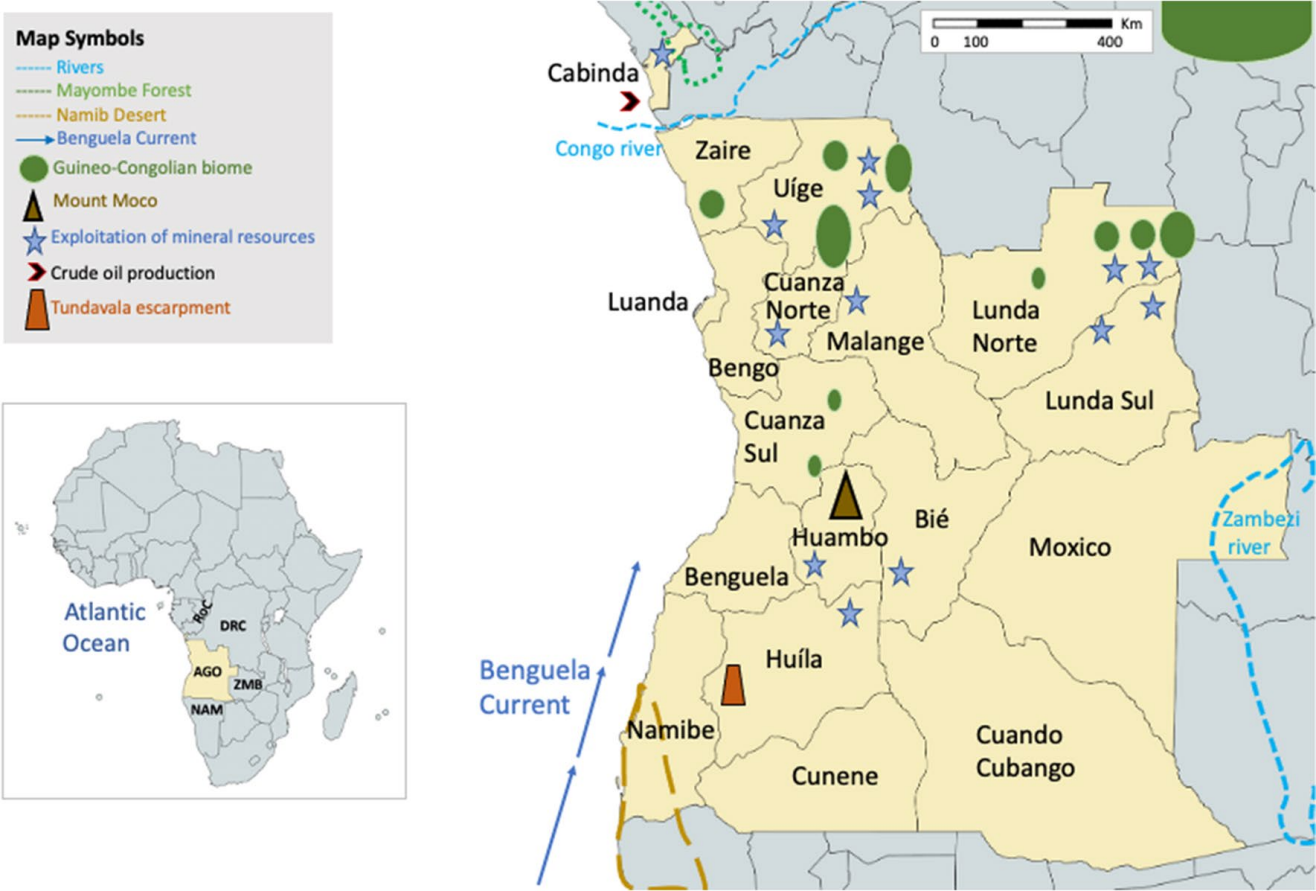
Angola and its provinces. Angola is located in the west coast of southern Africa, surrounded by the Republic of the Congo (RoC), the Democratic Republic of the Congo (DRC), Zambia (ZMB) and Namibia (NAM) (inset). The country is divided into 18 provinces (main map), including the northern-most Cabinda, which is separated from the lowest 17 provinces by an ocean-reaching sleeve of the DRC [46–49]

Angola, located on the southwestern coast of Africa, is the 7th largest country in the continent, with nearly 500,000 square miles. The country is divided into 18 provinces and shares borders with the Republic of the Congo (RoC) to the north, the Democratic Republic of the Congo (DRC) to the north and northeast, Zambia to the east and Namibia to the south (Fig. 3). The country encompasses both the southwestern edge of subtropical humid regions of Africa, in the north of the country, with tropical and subtropical forests, and the northern edge of the Namib Desert in Angola’s southwest, with arid areas of desert and steppe along its coastal and southern edges [37].

The Mayombe forest, Africa’s largest forest [38], spreads throughout Angola’s NW province of Cabinda as well as regions of the DRC, RoC and Gabon, fed by the Congo River, the second longest in Africa. Deforestation activities in the Mayombe region, and different economic activities including agriculture, mining and biofuel production, are linked with a potentially increased risk of malaria transmission [39–41]. The rainy season, from October to May, with temperatures of 28–33 °C and humidity near 100%, impacts malaria prevalence in the region, as these environmental factors play a major role in mosquito development and biting rates, as well as in the survival and development rate of the parasite [42–45].

Along the coast, the Atlantic ocean’s cold, northward-flowing Benguela current allows the coastal provinces to become relatively arid or semiarid due to substantial reduction in precipitation. In central-coastal provinces temperatures generally reach 20 °C, with a rainy and hot season extending from November to May, and a cool and relatively dry season (called *Cacimbo*) from July to late September [27, 28].

Angola’s low altitude coastal plains are separated from the interior by a steep escarpment that runs north to south across nearly the entire length of the country [48]. In the interior, Mount Moco, Angola’s highest mountain (peak at 2620 m), is located in Huambo province (Fig. 3). To the East of Mount Moco, a central plateau is marked by the west-central highlands reaching altitudes of 1600–1700 m. This plateau gives way to peneplains to the North and the whole eastern side of the country, mostly comprised of grasslands and savannas, as well as shrublands and woodlands [48]. Temperatures are cooler on the central plateau than along the coast, while in the far south sand dunes predominate, which give way to scrub forests [48].

This climate and ecological diversity, associated with an equally diverse range of soil and geological features, has resulted in the continent’s second most varied assembly of ecoregions in a single country [48]. The variation in rainfall (highest in the NE, along the border with the DRC, and lowest in the xeric SW), temperature, altitude and biome in turn affect the availability of suitable breeding grounds for the mosquitoes vectors [50–53]. In addition to rainfall, providing habitats for mosquito larvae, temperature (and possibly its fluctuations) are key factors in determining malaria transmission intensity [43, 54, 55]. Climate-based factors such as high precipitation and humidity, low altitude, land use and high temperatures, are the primary environmental determinants of malaria. Therefore, the importance of understanding the role of environmental factors provides opportunities for the design of highly targeted malaria control activities in the country.

### Malaria vectors in Angola

**Fig. 4:**
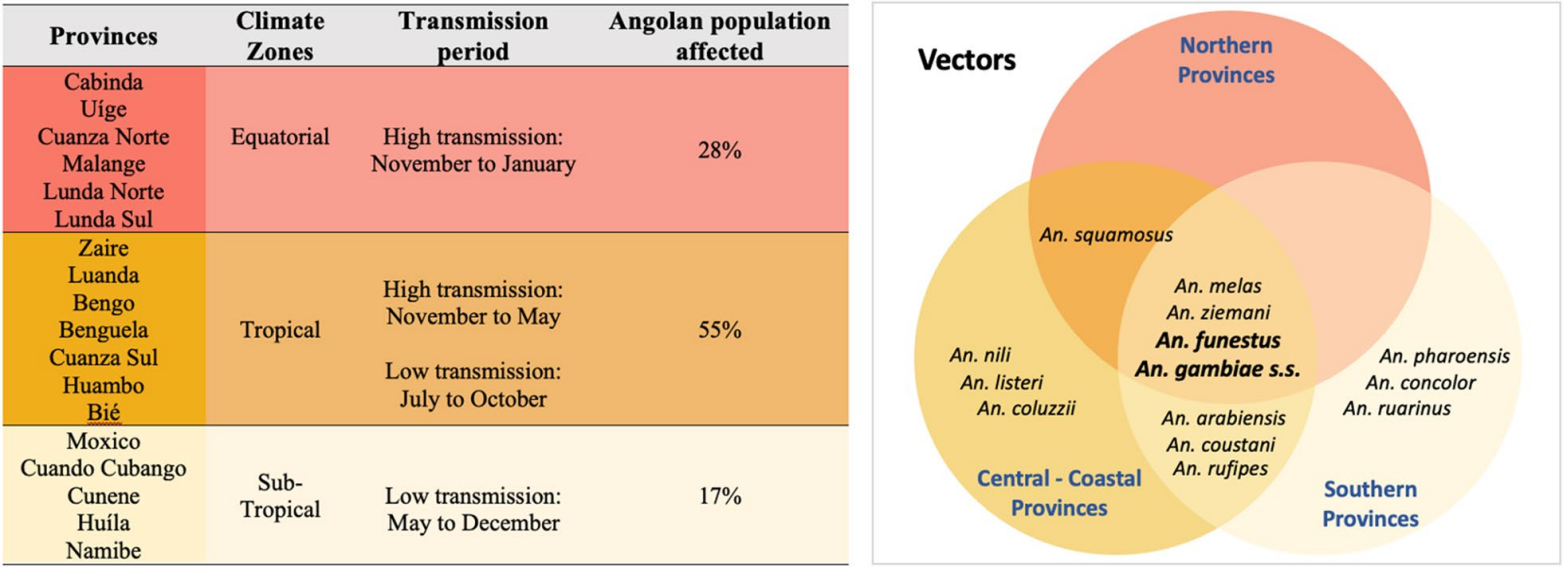
Dominant and secondary malaria vectors in Angola, by malaria endemicity region [27, 28]. Provincial stratification based on endemicity levels, from hyperendemic provinces (red), to mesoendemic with stable transmission (yellow), and mesoendemic with unstable transmission (beige) (as in Fig. 2A)

A key determinant of malaria transmission is the presence of vector species, which is constrained by environmental suitability [56]. Biomes described above are characterized by differences in rainfall, altitude, weather patterns, flora and fauna that all interact to produce variable habitability for *Anopheles* mosquitoes, which in turn influences the prevalence and burden of malaria across the country [57]. *Plasmodium falciparum* prevalence in Angola decreases from N and E to the SW, with a particularly high prevalence in the eastern province of Moxico and lowest prevalence recorded in the SW regions of Namibe, Huíla, Cunene and, to a lesser degree, Cuando Cubango [10], all patterns that mirror the distributions of rain fall, mosquito prevalence and malaria incidence in the country (Fig. 4) [10, 48, 58].

The information on individual *Anopheles* vector species in Angola is relatively scarce, possibly due to a loss in expertise and disruptions to studies and to control interventions throughout Angola’s war for independence and subsequent civil war, posing a major challenge to Angola’s NMCP [57]. In the early 2000s, a few studies were performed on the spatial distribution of mosquito vectors, showing that main vectors in Angola included members of the *Anopheles gambiae* species complex (particularly *An. gambiae *sensu stricto (*s.s*.), *Anopheles arabiensis* and *Anopheles melas*) and *Anopheles funestus* [27, 28]*.* The available information shows the geographical distribution of *Anopheles* species to be heterogenous. *An. gambiae s.s.* and *An. funestus* are among the most widespread vectors [57, 59], although *An. gambiae* appears to predominate across the forested hills in the northern provinces (Zaire, Malange and Uíge), whereas *An. funestus* predominates in Central, Southern and some coastal regions [57, 60, 61]. *Anopheles melas* is most frequently found in coastal areas, and *An. arabiensis* and *Anopheles pharoensis* in the southern and central highland provinces (Huambo, Benguela, Cunene, Huila and Namibe) [16, 62, 63]. *Anopheles pharoensis* and *Anopheles coustani*, historically documented as playing a minor part in malaria transmission, were considered secondary vectors [27, 28, 57]. Recently, a growing number of comprehensive field studies undertaken across the country, including USAID/PMI-supported entomological evaluations in collaboration with the Government of Angola, demonstrated the existence of additional secondary malaria vectors. These included *Anopheles nili s.s, Anopheles squamosus, Anopheles ziemanni, Anopheles listeri*, *Anopheles concolor* and *Anopheles ruarinus* in Angola’s central and southeast regions, with the latter two in particular being quite rare (Fig. 4) [59, 64].

The observed differences in distribution are in part seasonal, possibly associated with rainfall and/or humidity. For example, in the coastal province of Bengela, *An. gambiae* was found to predominate in the wet season, while in the dry season *Anopheles coluzzii* was most frequent, together with *An. melas* and *An. listeri* [65]. The ecosystem and its associated flora and degree of human intervention also impact vector distribution. For example, while *An. nili s.s.* is found across almost all forested areas of sub-Saharan Africa, *An. gambiae* and *An. arabiensis* prefer to breed in semi-open and open areas impacted by human activities, such as agriculture, deforestation, and mining [66].

Even though broad distribution patterns of the different vectors are apparent, fine scale geographic distribution and the relative contributions of different vectors to malaria transmission are still poorly described and will greatly benefit from the ongoing studies currently under way, in initiatives prioritized by the USAID/PMI and/or supported by the E8 initiative [21, 61].

### Malaria control and prevention in Angola

#### Prevention

In recent years, the Government of Angola, together with several international partners, has invested in several malaria prevention interventions targeting both disease vectors and the parasite. Implementation of intermittent preventive treatment of malaria in pregnancy (IPTp) was initiated in 2006 [67] and, by 2019, roughly 10 million Angolans (~ 30% of the population) had access to insecticide-treated bed nets (ITNs), including long-lasting insecticidal nets (LLINs), or indoor residual spraying (IRS) [10]. Between 2016 and 2020 the NMCP in Angola aimed to reduce malaria cases across the country by 60% relative to 2012, including a concerted focus of eliminating malaria in two provinces to the south and east (Cunene and Cuando Cubango) [24, 68]. Accordingly, a community-centred pilot project called ADECOS was deployed between 2017 and 2019. Under this project, community development and sanitary agents, linked to municipal health centres, received 3 months of training and were responsible for promoting education related to sanitation, health and water, and for providing basic health services to local communities, including the use of rapid diagnostic testing (RDTs) and treatment for confirmed uncomplicated malaria with artemisinin-based combination therapies (ACTs). Under this project, > 100,000 RDT were performed and nearly 40,000 malaria cases were diagnosed and treated [69].

Despite considerable reduction in malaria incidence across the south during this period (Fig. 2A), the broader goal of malaria reduction countrywide had limited success. Consequently, starting in 2021, the Angolan Ministry of Health, in partnership with the USAID/PMI, has focused interventions in six contiguous, mostly hyperendemic, northern provinces, namely Cuanza Norte, Lunda Norte, Lunda Sul, Malange, Uíge and Zaire. These interventions include increasing ITNs accessibility to the population through routine channels (antenatal care clinics and the expanded programs for immunization) and mass campaign distributions every 3 years [70]. Through this partnership, Angola’s National Strategic Plan towards malaria control aims to reduce malaria morbidity and mortality by 40% and 50%, respectively, by 2025, through initiatives that include reaching at least 80% of the Angolan population with one or more malaria control intervention, including larvicide, LLINs and/or IRS [16, 21, 71].

#### Diagnosis and anti-malarial drug policy

By the early 1980s, chloroquine resistance had spread throughout Africa [72], which resulted in chloroquine’s removal as a first-line treatment for uncomplicated malaria in Angola and was replaced by amodiaquine (AQ) or sulfadoxine/pyrimethamine (SP) [28, reviewed in 67]. Starting in 2004, with increasing evidence of emergent SP resistance [73], Angola adopted the use of ACTs for the treatment of uncomplicated malaria, a switch implemented country-wide by 2007/8 [67].

In 2006, the Angolan NMCP introduced SP intermittent preventive treatment among pregnant women (IPTp) [28, 67]. Since the use of ACTs as the first-line treatment became widespread throughout the country, the potential presence of drug resistant parasites has been monitored in a few hyper- or mesoendemic malaria provinces by assessment of the frequency of drug resistance markers [74, 75] and/or with drug efficacy trials [76–79]. Even though these monitoring efforts have found no evidence of mutations in *kelch*13 known to be associated with artemisinin resistance in SE Asia [75, 76, 80, 81] or resistance to artemisinin derivatives [79], the continued presence of mutations in *dhfr* and *dhps* is consistent with resistance to SP and mutation in *mdr1* and *pfcrt* are consistent with resistance to lumefantrine and amodiaquine [75, 79]. In addition, markers associated with resistance to lumefantrine, first observed in 2013 [77], may continue to reduce the efficacy of artemisinin-based combinations using this partner drug [76, 78]. Finally, additional challenges to successful treatment include the availability in counterfeit anti-malarial drugs, self-diagnosis and self-medication, and non-compliance with drug administration schedules [82]. On the whole, improvements of routine malaria information systems, development and updating national malaria treatment policy, with a switch to ACTs, and monitoring anti-malarial drug resistance across the country may all have contributed to a reduction of malaria deaths around 2005, and a constant decline in the per capita malaria mortality and incidence rates [28] (Fig. 1). It is unknown whether these control measures have had a significant impact on parasite prevalence.

#### Vector surveillance and control

Given the NMCP’s strategic goals and increased commitment to advancing vector monitoring and control activities, interventions have focused on routine and mass distribution of LLINs or ITN, particularly in areas where IRS was previously implemented, and IRS in selected municipalities [21, 71]. Broad distribution of nets and IRS in southern provinces of Cuando Cubango and Cunene activities, together with ADECOS, also aimed at supporting Namibia’s malaria pre-elimination efforts through cross-border initiatives [30]. Following ITN distribution campaigns in 2017–2019, ITN coverage increased from 9.2% to 98% in Cuando Cubango. In the neighbouring province of Cunene coverage reached similar levels, although a year later coverage estimates were down to 63%, indicating that new ITNs/LLINs must be continuously supplied to replace those that have lost efficacy and are torn, in order to maintain high levels of coverage [30].

In recent years, entomological surveillance, assessment of insecticide resistance and capacity building through research infrastructure and personnel training, especially in hyperendemic areas, together with logistics associated with procurement and distributions of ITNs and LLINs, has been a priority of USAID/PMI [21, 70]. In southern provinces, some of these activities have been the purview of the Angolan Government and international partners. For example, from November 2020 to January 2021, the Angolan NMCP and collaborators, including E8 partners, Global Fund and the MENTOR Initiative, conducted an entomological surveillance study to identify drivers of transmission, and determine levels of insecticide resistance in Cuando Cubango municipalities where IRS had been previously implemented [60, 64]. Among surveyed sites, *An. funestus *sensu lato (*s.l.*) was confirmed as the most abundant and widely distributed mosquito vector (prevalence > 90%); *An. rufipes* and *An. gambiae s.l.* were widespread but rarer (< 5%), with 100% susceptibility to the organophosphate and pyrethroid insecticide classes among the latter [64]. The development of long-term sustainable surveillance measures that inform the geographic and temporal distribution of anopheline mosquitoes and their relative abundance remain a priority for evidence-based malaria vector control programmes.

### Spread of malaria in Angola

#### Internal population displacement

Human migration is one of the main drivers of spread of *P. falciparum* [83–85], making human movement across Angola an important factor to consider in understanding the dynamics of malaria in the region. Of the current population of ~ 36 million, about two thirds live in urban centres, including 2.8 million in the capital city, Luanda, and 9.1 million in the overall Luanda province [86]. Migration to Luanda grew rapidly in the end of the twentieth century, due to armed conflicts elsewhere, as Luanda was considered a safer province compared than the rest of the country. An estimated 4.1 million people were displaced internally during the 27 year-long civil war, which ended in 2002 [87, 88]. Since the turn of the century, and once the war was over, Angola’s strong economic boom continued to stimulate migration within the country to large population centres, especially the capital [89, 89].

Currently, the number of refugees and internally displaced Angolans is a small fraction of what was observed over two decades ago, and is mostly due to natural disasters, such as floods [90]. However, southern Angola is experiencing its most severe drought in 40 years, which has now lasted for more than 3 years, which triggered an acute food insecurity alert, in September of 2021, by the Integrated Food Security Phase Classification (IPC) initiative and produced growing fears of widespread famine and population displacement, as well as cross-border migration into Namibia [91, 92]. It is critical to closely track the situation, to ensure availability of needed public services, including early detection and treatment of malaria [93]. Finally, since migrants may carry malaria parasites across provinces, as well as across international borders, with associated challenges for control and elimination, the identification of the most commonly used routes and destinations of human migration within the country can help inform public health interventions aimed at controlling the disease.

#### Malaria among Angola’s neighbours and the elimination eight initiative (E8)

**Fig. 5:**
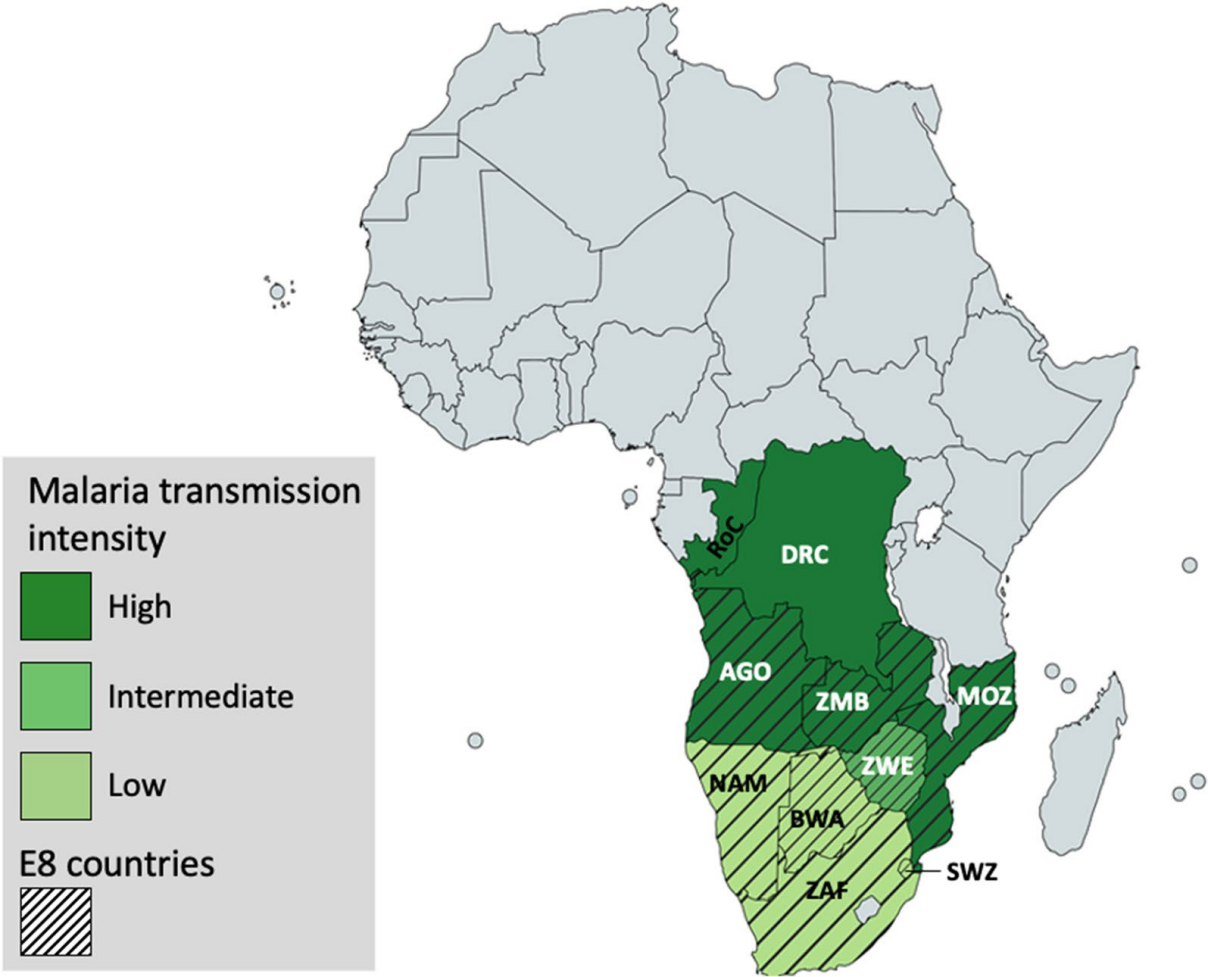
Angola’s neighbouring countries and Elimination 8 (E8) front-line countries. Geographic zones of varying malaria epidemiology distribution in Angola’s neighbouring countries and key partners of the Elimination 8 (E8) initiative (*AGO* Angola, *NAM* Namibia, *ZAF* South Africa, *BWA* Botswana, *ZWE* Zimbabwe, *ZMB* Zambia, *MOZ* Mozambique, *SWZ* Eswatini, *DRC* Democratic Republic of the Congo, *RoC* Republic of the Congo). The spatial distribution of malaria transmission is stratified as low (light green), medium (medium green) and high (dark green) [10]

The movement of people across international borders poses a critical challenge to malaria control due to properties that are often unique among border regions, where disparate resources, policies and challenges across borders makes it exceedingly difficult to achieve uniform success [94, 95]. Angola shares > 3300 miles with its neighbours (Fig. **5**) including borders with the RoC and DRC, countries that have hyperendemic malaria transmission and similar climatic and ecological conditions to northern Angola [10]. Challenges to malaria control in these countries are varied, from agriculture-associated vector breeding grounds and farm worker migration, to lack of sanitation infrastructure, and population displacement and migration associated with socio-political instability [96–99]. In contrast, southern Angola is bordered by countries with lower malaria prevalence, and migration out of Angola may contribute to the spread of malaria in neighbouring countries [22, 66]. Such may be the case in Namibia which, despite the country’s successful reduction in malaria burden over the past decade resulting low transmission rates, since 2016 it has experienced several outbreaks in its northern region [100].

These challenges have led to the emergence of geographically-based initiatives to tackle malaria in border regions [23, 101–103]. The Elimination Eight Initiative (E8) (https://malariaelimination8.org/) is a coalition of eight countries within the Southern African Development Community (SADC) whose aim is to collaboratively work towards malaria elimination in the region by 2030 **(**Fig. 5**)** [104]. The E8 is pioneering an ambitious regionally-based strategy including cross-border initiatives to roll back malaria from the southernmost countries, including Angola, and accelerate collective progress towards zero transmission [103, 104]. As part of E8 initiatives, eight border malaria health posts and surveillance teams were deployed to the border areas of the provinces of Cuando Cubango and Cunene, where “Test, Treat and Track” (TTT) measures were implemented, providing access to malaria diagnosis and treatment services, specifically targeting two key populations at risk of malaria, namely underserved residents of border districts, and mobile and migrant populations. Since becoming operational, these malaria health borders have tested and treated thousands of malaria cases. Among the major E8 accomplishments over the past decade is the reduction of malaria by more than 30% in border regions of participating countries [104]. Nevertheless, E8 initiatives in Angola target only the southern and SE borders, leaving initiatives aiming to control malaria in the regions of the country with higher and stable malaria transmission to the Angolan government and other international partners.

#### The potential of *P. falciparum* demography studies in Angola

It is currently unknown if the *P. falciparum* population in Angola is panmictic or structured, and if the latter, the extent of its fragmentation. This knowledge is critical to inform successful malaria control initiatives. For example, it is unknown if Angola’s *P. falciparum* population is fragmented, with parasites persisting year-round in regions of low transmission as subpatent infections in asymptomatic carriers, or if instead the Angolan *P. falciparum* population behaves largely as a single, panmictic population, with hyperendemic regions with year-round transmission sourcing parasites to regions of lower or epidemic transmission. Accurate characterization of the structure and dynamics of the *P. falciparum* population is essential to develop targeted interventions to control malaria and prevent its spread. If hyperendemic malarious regions are responsible for disseminating parasites to regions of low malaria prevalence, it is imperative to curb malaria at the source. Alternatively, if *P. falciparum* is able to persist year-round in regions of low endemicity, it is critical to identify and treat asymptomatic carriers [105, 106].

The movement of internally displaced people and economy-driven population migration could contribute to eliminate natural barriers that exist between potential parasite subpopulations, for example those imposed by differences in climate. A thorough characterization of the existing population structure of *P. falciparum* in the country will inform the extent to which molecular markers of different populations can be identified and help determine if and how future human population displacement fuels the spread of the parasite across provinces.

It is also unclear if differences in the level of endemicity across Angola affect infection properties. Broadly speaking, in countries of high transmission intensity, infections are often polyclonal and parasite populations are highly diverse and panmictic [107, 108]. In contrast, at the edges of the malaria distribution or in regions where malaria is epidemic, the parasite population is fragmented, infections often contain a single genotype, and clonal expansion is more common [85, 109, 110]. Across Angola these two extremes, as well as variations in between, can all be found. An in-depth molecular epidemiological study of malaria infections from Angolan provinces of different endemicity remains to be done, which would reveal whether these general patterns are observed in Angola, and improve understanding of malaria transmission in the country.

The presence of genetically distinct *P. falciparum* populations in east and west African countries is well established, contributing to the high genetic diversity of *P. falciparum* in Africa [111, 112], and the most comprehensive study of *P. falciparum* genetic variation in Africa to date demonstrated the existence of several genetically distinct parasite populations south of the Sahara [85]. Angola, which encompasses the southwestern-most edge of the African *P. falciparum* distribution, is surrounded by distinct *P. falciparum* populations; one, in Central Africa, is represented by parasites from Gabon and Cameroon, and another, in south-central Africa, is composed of parasites from the DRC [85]. Countries surrounding Angola in Central Africa might therefore hold the key to understanding *P. falciparum* dynamics in the north and eastern edges of Angola (Fig. 5). However, due to dispersed and limited information reflecting malaria situation in Angola, and the lack of genetic or genome-wide data from the country, it is still unknown how distinct its *P. falciparum* population is in relation to those of its neighbours, and hence whether parasite genetic variation has the potential to inform the directionality and intensity of malaria transmission between countries.

Finally, collection of genomic data can help monitor and identify mutations in genes associated with drug resistance and infer the potential for delayed or failed treatments of Angola’s current frontline therapy, artemether-lumefantrine. From these data, the Angolan NMCP can identify areas where malaria interventions would have the greatest impact and progress towards malaria elimination. Identifying such areas is critical in a resource-limited environment and the prioritization of where needs are greatest needs to be accounted for. In the future, using mobile applications integrated with genomics signals into decision-making outputs could offer valuable information to the international agencies and malaria control program managers**.**

## Conclusion

Angola continues to adapt its approach within a challenging and variable environment by implementing robust strategies aligned with neighbouring NMCPs to synchronize efforts and reach the ambitious goal of malaria elimination by 2030. Over the previous decade, Angolan efforts have decreased malaria incidence, but complex factors threaten the sustainability of this progress. Diverse environments, transmission intensities, mosquito vectors as well as internal population displacement and emerging of resistance to drug therapies and insecticides are some of the challenges Angola faces, which make prioritization difficult.

Both internal agencies and stakeholders, including the Angolan NMCP and regional governments, and external partners such as the WHO and PMI, agree that malaria control and elimination are top priorities for the Angolan Government. However, all parties contend that increased funding, improved planning of resource acquisition and management, and, in particular, a larger and better trained healthcare workforce are critical for the sustained reduction of the malaria burden to the country [113]. Ultimately, the Angolan NMCP requires continued support in the form of traditional malaria control methods, as well as support to implement novel techniques to expedite decreasing trends of malaria burden. Although malaria remains a challenging public health burden in Angola, the progress made by the NMCP via within-country and cross-border interventions in the past two decades is significant and through continued effort and support could lead to permanent gains.

## Data Availability

Not applicable.
